# Goal Achievement and Academic Dropout Among Italian University Students: The Mediating Role of Academic Burnout

**DOI:** 10.3390/ejihpe15010003

**Published:** 2025-01-06

**Authors:** Arianna Nicita, Angelo Fumia, Concettina Caparello, Carmelo Francesco Meduri, Pina Filippello, Luana Sorrenti

**Affiliations:** 1 Department of Health Sciences, University Magna Graecia of Catanzaro, 88100 Catanzaro, Italy; arianna.nicita@unicz.it (A.N.); concettina.caparello@unicz.it (C.C.); carmelofrancesco.meduri@unicz.it (C.F.M.); 2 Department of Clinical and Experimental Medicine, University of Messina, 98124 Messina, Italy; giuseppa.filippello@unime.it (P.F.); luana.sorrenti@unime.it (L.S.)

**Keywords:** goal achievement, academic burnout, academic dropout, Italian university students

## Abstract

As stated by the Goal Orientation Theory, students want to achieve a goal for multiple reasons, with each having a different impact on academic performance. This framework encompasses a three-factor model of goal achievement: a mastery goal, a performance-avoidance (PAv) goal, and a performance-approach (PAp) goal. Students may experience elevated stress levels and burnout due to adopting an ineffective approach to goal achievement. This can lead to a loss of interest in studies and even physical and psychological exhaustion. In severe cases, this may result in students abandoning their studies early. This study aims to integrate these factors into a comprehensive model. A cross-sectional study comprising 1497 Italian university students examined the mediating role of academic burnout (professional efficacy, cynicism, and emotional exhaustion) in the association between achievement goals (mastery, PAv, and PAp goals) and the intention to drop out (ID). The questionnaires were administered from October 2022 to September 2023. Structural equation modeling was employed to evaluate the association between variables. The results of the mediation analysis indicate that cynicism and professional efficacy fully mediate the association between mastery and dropout. Cynicism (β = −0.28, *p* < 0.001) and professional efficacy (β = −0.17, *p* < 0.001) were both negatively associated with ID, while they partially mediate the association between PAv goals and ID (cynicism: β = 0.21, *p* ≤ 0.001; professional efficacy: β = 0.05, *p* ≤ 0.001), and between PAp goals and ID via professional efficacy (β = −0.04, *p* ≤ 0.001). This study contributes to the currently limited literature on the relationship between achievement goals, burnout, and ID in a sample of university students. The findings of this study may have useful implications for the application of interventions that impact students’ well-being and academic success, potentially limiting their possible dropout.

## 1. Introduction

The well-being of university students has recently emerged as a prominent topic in academic research, reflecting a growing awareness of the multifaceted challenges they confront during their academic pursuits (Wissing et al., 2011). In this context, students may encounter situations that have a strong emotional impact, which can affect their learning experience (Mega et al., 2014). A significant concern is the likelihood of these students being exposed to negative situations, such as academic failure, which may result in a loss of interest and demotivation to continue their studies (Bresó et al., 2011; Mason & Nel, 2012; Mostert & Pienaar, 2020). The existing literature has investigated the potential factors that may either foster or hinder success but has not yet provided an integrated view of the influence of these variables on the academic path (Abreu Alves et al., 2022; Altharman et al., 2023; Fazli & Fouladchang, 2019; Mostert & Pienaar, 2020; Nadon et al., 2020; Poorgholamy et al., 2020; Usán Supervía & Salavera Bordás, 2020). During their educational experience, motivation acts on students as a driving force that energizes, directs, and sustains students’ engagement in learning activities, influencing their self-regulation, academic persistence, and goal achievement (Boekaerts, 2010).

The purpose of the student in pursuing goal achievement has been thoroughly studied within the framework of the Goal Orientation Theory (Ames, 1992; Atkinson, 1964; Elliot, 2005), which analyzes the role of students’ academic motivation in the learning process and explores adaptive and maladaptive behavioral patterns in relation to academic successes and failures (Dweck, 1986; Kaplan & Maehr, 2007; Veroff, 1969). Goal orientation is defined as the efforts and actions required to achieve a goal. These goals are the outcome that a person is trying to accomplish and are characterized by the needs, values, and situational patterns specific to each learner. They are considered the driving force behind students’ engagement and participation in learning activities (Nicholls, 1984; Pintrich, 2003). This motivational approach helps us understand how learning behavior is generated, initiated, and directed (McClelland, 1961) and *why* and *how* students can engage in specific learning behavioral outcomes (Elliott & Dweck, 1988). Dweck (1986) explained that students may be oriented toward either *mastery* or *performance*. Mastery-oriented students attempt to become more competent to increase their competence, while performance-oriented students are more focused on seeking confirmation of their competence. Mastery-oriented students exhibit a greater focus on their learning process, demonstrating increased autonomy and self-regulation, which often leads to positive outcomes (Kaplan & Maehr, 2007). They mainly prioritize personal development, which is associated with higher levels of self-efficacy, self-regulation, problem-solving, and well-being; better academic achievement; good active coping strategies; and proactive social behavioral patterns (Bereby-Meyer & Kaplan, 2005; Dweck & Leggett, 1988; Dykman, 1998; Elliot, 1999; Elliott & Dweck, 1983; Graham & Golan, 1991; Kaplan et al., 2002). Mastery goals are connected with an implicit incremental theory of intelligence and are pursued by students who are willing to improve their skills and competencies iteratively, not being excessively concerned when facing failures or low performance levels during the learning process (W. C. Liu, 2021). Conversely, performance-oriented students are often characterized by maladaptive behavioral, emotional, and cognitive patterns (Ames, 1992), which can lead to helplessness, and are characterized by avoidance, the manifestation of negative symptoms (anxiety, fear, etc.), and the tendency to show negative self-perceptions (Dweck, 1986).

Starting from this dichotomous model, Elliot and Church (1997) analyzed the relationship between different achievements and the motivational drive underlying each action, identifying a three-factor model of goal achievement: a mastery-achievement goal, a performance-avoidance (PAv) goal, and a performance-approach (PAp) goal. Students with a PAv orientation are motivated by the need to avoid demonstrating low ability and are more likely to elude situations in which they could fail. PAv goals are associated with self-sabotaging and maladaptive behaviors, such as anxiety-related issues, test anxiety, lower levels of learning self-efficacy, and lower well-being (Church et al., 2001; Darnon et al., 2009; Elliot, 1999; Elliot & Church, 1997; Elliot & Harackiewicz, 1996; Peetsma & Veen, 2013; Sideridis, 2005; Vandewalle et al., 2019). Conversely, students with a PAp orientation are motivated by the desire to demonstrate high ability and competence to others and are driven by the pursuit of success. This model was further parceled out, arriving at the level of the 2 × 2 Goal Orientation Theory, which realizes a framework based on a four-factor model, where the mastery goal construct is also divided into two different types: mastery-approach and mastery-avoidance goals (Elliot, 1999). Students with a mastery-avoidance orientation engage in a task to prevent losing previously acquired knowledge or abilities (deskilling); specifically, it is *the goal to avoid doing worse than one has done before* (Van Yperen et al., 2009) and may lead to anxiety and lower performance (Howell & Watson, 2007; Hulleman et al., 2010). Despite this, the trichotomous model persists as the most widely utilized model, primarily due to the paucity of a comprehensive analysis of the mastery-avoidance orientation (Cellar et al., 2011; Elliot, 1999; Kaplan & Maehr, 2007; Linnenbrink & Pintrich, 2000; Pintrich, 2003; Vandewalle et al., 2019).

The present framework was developed primarily on the basis of data from high school students. Therefore, there is a lack of empirical research that has applied this theory to the problems that university students encounter in their course of study. Nevertheless, the literature has shown that students with a mastery orientation from the earliest levels of education showed greater academic well-being and adaptive learning patterns than those with an avoidance orientation, even at university. Mastery orientation is also more effective among older students (young adults) than younger students (Utman, 1997). Conversely, performance-oriented students, regardless of the level of learning acquired, strive to achieve high scores and frequently exhibit maladaptive behaviors (Tuominen-Soini et al., 2012), especially in the emerging adult stage. This period of life is permeated by different transformations in which students are often subjected to different stressful situations, presenting the main symptoms of academic burnout (Schauffeli & Salanova, 2007).

Burnout is characterized by emotional, physical, and mental exhaustion (Schaufeli & Greenglass, 2001) and can affect individuals throughout their life contexts (IsHak et al., 2013). Originally, research on burnout focused on the work context. However, burnout has also been found in other types of non-work-related settings, such as among students in academic contexts (Bianchi et al., 2014; Chambel & Curral, 2005; Noh et al., 2013; Shin et al., 2011). Academic burnout acknowledges that students, although not traditional workers, undergo structured events in their studies that resemble work-related tasks. For example, taking exams, attending classes, and completing assignments are all part of students’ academic activities and can be considered “work” (Shin et al., 2011). In particular, in their academic careers, some students may develop disinterest, a lack of commitment, and a sense of inability due to various stressful situations (Maslach, 2001), such as piles of homework, insufficient sleep, unhealthy eating patterns, concurrent demands from the family, inadequate or insufficient physical activity, inadequate time management, and unrealistic goals (Liu et al., 2023). Academic burnout is a three-dimensional construct that comprises emotional exhaustion, cynicism, and professional efficacy. Emotional exhaustion, characterized by low emotional output and attrition, is correlated with physical exhaustion, such as fatigue (Caballero et al., 2015). Students who run out of resources become emotionally and physically exhausted and find it difficult to accomplish their educational responsibilities. Exhaustion can also result in the inability to put effort into or show enthusiasm in tasks related to their studies (such as finishing coursework or revising), which makes their performance appear poorer when their work or competency is assessed (Liu et al., 2023). Cynicism, also called depersonalization, manifests in students who show indifference and little interest in learning. This phenomenon may cause students to distance themselves from the classroom, teachers, and work (Madigan & Curran, 2021). This will probably result in students missing important information, not taking advantage of opportunities to obtain support, and generally avoiding studying time. These behaviors lead to lower academic achievement compared with students who exhibit less cynicism (IsHak et al., 2013). Professional efficacy reflects students’ self-awareness of their abilities (Schauffeli & Salanova, 2007). High professional efficacy is linked to feelings of productivity and effectiveness, while students with low efficacy are more likely to engage in avoidance behavior. This may contribute to perceptions of reduced accomplishment, which may lead to reduced achievement (Madigan & Curran, 2021).

Situations characterized by high stress may lead to emotional exhaustion, cynicism, and lower levels of professional efficacy, which could result in students gradually becoming detached from the academic context. These factors represent crucial variables in the academic path, as students who adopt maladaptive behaviors often lose interest because of negative experiences (Fiorilli et al., 2017; Salmela-Aro & Vuori, 2015). This may result in a reduction in motivation and engagement in university, which may in turn lead to a sense of estrangement and, in the most severe cases, in students dropping out of their studies (Caballero et al., 2015).

Dropout in higher education is a multifaceted phenomenon and a longitudinal process that causes students to leave university or school without finishing their studies (Almeida et al., 2019). Student dropout rates are a global concern, transcending geographical boundaries and demographic characteristics (Lorenzo-Quiles et al., 2023; OECD, 2019). Recent statistics at the European level have identified that, in 2023, approximately 9.5% of young people between the ages of 18 and 24 have discontinued their education and training prematurely. Italy, in particular, exhibits a higher dropout rate than the European average, with a 2023 percentage of 10.5% (*Early leavers from education and training*, 2024). The relationship between the frequency of thoughts about dropping out of academics and the actual decision to drop out has been widely studied in educational research (Abdulghani et al., 2023; Morelli et al., 2023; Tayebi et al., 2021). A key finding is that thoughts about dropping out are not simply passing considerations but are part of a larger process of disengagement. Aarkrog et al. (2018) describe this process as fragmented and influenced by various factors, indicating that dropout decisions develop over time rather than being a sudden choice (Aarkrog et al., 2018). This perspective is further substantiated by Cobo-Rendón et al.’s (2023) characterization of these thoughts as manifestations of apathy and disengagement from academic responsibilities (Cobo-Rendón et al., 2023). The reasons behind academic dropout can be attributed to the characteristics that students possess at the time of enrollment in higher education. These include the quality of their primary education and study skills, the level of commitment to the course (goal achievement), and social aspects, which are connected to interaction and interpersonal integration and effort, indicating the motivational level of the student. Additional factors include how students handle their academic performance and how much their physical and mental health is compromised (Almeida et al., 2019; Ambiel, 2015). In the academic career, goal achievement has emerged as a significant predictor of this phenomenon, reflecting students’ attitudes toward their academic pursuits (Aarkrog & Wahlgren, 2022). Students with a mastery goal orientation perceive study abilities as something they can acquire via effort. They also favor challenging assignments and are tenacious when faced with difficulties (Bzuneck & Boruchovitch, 2019; Dalbosco et al., 2018; Senko et al., 2011). These attributes reduce institutional issues in the academic environment, which lowers the intention to drop out (ID) among students who pursue this goal (Bardach et al., 2020). In contrast, students with a PAv goal orientation are driven to avoid low performance. The anxiety associated with being classified as a low-achieving student has been observed to influence student behavior. Students with a PAp goal orientation endeavor to distinguish themselves from their peers as they rely on external validation to attain a sense of accomplishment (Bzuneck & Boruchovitch, 2019; Senko et al., 2011). High levels of anxiety are associated with both performance goals, and students with this orientation may also be at an increased risk of dropping out of university (Bzuneck & Boruchovitch, 2019; Dalbosco et al., 2018; Senko et al., 2011). Therefore, some students may not possess the necessary strategies and competencies to successfully cope with the demands of academic life, thus developing a negative attitude toward education, losing interest in their studies, and even experiencing physical and psychological exhaustion (i.e., burnout) (Elliot & Church, 1997; Fazli & Fouladchang, 2019; Nadon et al., 2020; Poorgholamy et al., 2020; Usán Supervía & Salavera Bordás, 2020). This may lead them to abandon their studies early (Usán et al., 2019). The literature suggests that students’ goal orientation plays a crucial role in making students feel overwhelmed and drained by the continuous demands of their studies or to lose interest in their academic pursuits, questioning the value of their education. Therefore, goal orientation may predict the rise of academic burnout (Elliot & Church, 1997; Fazli & Fouladchang, 2019; Nadon et al., 2020; Poorgholamy et al., 2020; Usán Supervía & Salavera Bordás, 2020). If the burnout situation persists over time, this may promote premature dropout of the course of study before its completion (Mostert & Pienaar, 2020). Compared to previous research that has individually examined the relationships between goal orientation and academic burnout (Fazli & Fouladchang, 2019; Nadon et al., 2020; Poorgholamy et al., 2020; Usán Supervía & Salavera Bordás, 2020) and the intention to drop out and academic burnout (Abreu Alves et al., 2022; Altharman et al., 2023; Mostert & Pienaar, 2020), this study aims to integrate these factors into a comprehensive model, thus offering a broader and deeper view of the dynamics of these constructs among Italian university students. A structural equation model (SEM) with latent variables was employed to evaluate the relationship between these variables. Based on the trichotomous model of the Goal Orientation Theory (Elliot & Church, 1997) and previous research that has focused on academic burnout with dropout intentions (Mostert & Pienaar, 2020), it is expected that goal orientation (mastery goals, PAv goals, and PAp goals) will be directly related to academic burnout (emotional exhaustion, cynicism, and professional efficacy) and that there will be an indirect relationship with the intention to drop out. Academic burnout is expected to fully mediate the relationship between goal orientation and academic dropout intentions.

## 2. Methods

### 2.1. Participants

The study sample consisted of 1497 Italian university students, including 1043 females (69.7%), 418 males (27.9%), and 36 students of unspecified gender (2.4%). All participants ranged in ages from 18 to 30 years of age (M = 23.80; SD = 2.99) coming from different regions of Italy, including Northeast Italy (4.3%), Northwest Italy (8.4%), Central Italy (10.3%), Southern Italy (66.4%), and the islands of Italy (10.6%), with a percentage of 59.8% of off-campus students (students who, in order to attend university, temporarily move to a city other than their usual place of residence or home town). The participants were recruited from various degree courses, such as healthcare (12.0%), medical (17.5%), scientific–technological (25.7%), and humanities–social (44.9%), and from various levels of the degree course. The sociocultural level of the students was also analyzed based on the highest educational attainment of their parents. The students were divided into two main categories: 26.8% were classified as having a low sociocultural level (parents obtained an elementary or middle school license), while the remaining 73.2% were categorized as having a medium-high to high sociocultural level (parents obtained a diploma or a degree).

### 2.2. Instruments

A demographic questionnaire collected the participants’ basic demographic information, including age, gender, and region of origin. Furthermore, information about the academic path was collected (whether the student was away from home or not, the university course of study, and the level of the degree course).

For this study, the Patterns of Adaptive Learning Scale (PALS; Midgley et al., 2000) was used to assess students’ achievement goals and other motivational constructs within educational environments. This scale consists of 17 items that assess personal achievement goal orientations, including 6 items for the Mastery Goal Orientation (e.g., “I like class work that I’ll learn from even if I make a lot of mistakes”), 6 items for the Performance-Avoidance Goal Orientation (e.g., “It’s very important to me that I don’t look stupid in my class”), and 5 items for the Performance-Approach Goal Orientation (e.g., “Doing better than other students in class is important to me”). Participants indicate their responses on a 5-point Likert scale ranging from 1 (not true at all) to 5 (strongly true). The reliability and validity of the Italian version of the PALS have been demonstrated in a previous study (Alivernini et al., 2018).

The Maslach Burnout Inventory Student Survey (MBI-SS; Maslach et al., 1997) was used to assess academic burnout. The MBI-SS comprises three scales totaling 15 items: emotional exhaustion (5 items, e.g., “I feel used up at the end of a day at university”), cynicism (4 items, e.g., “I doubt the significance of my studies”), and professional efficacy (6 items, e.g., “During class, I feel confident that I am effective in getting things done”). Each item is assessed on a 6-point Likert scale ranging from 0 (never) to 6 (always). The reliability and validity of the Italian version of the MBI-SS have been demonstrated in a previous study (Portoghese et al., 2018).

The intentions of students to continue or, conversely, to abandon their academic path were measured using questions derived from the Intentions to Persist Versus Drop Out scale (Hardre & Reeve, 2003). In the original version, the authors, drawing on Vallerand, Fortier, and Guay’s work (Vallerand et al., 1997), assessed students’ intention to persist or abandon their studies. The scale is made up of 4 items. In the present study, students were asked how often they “think about leaving university to do something different”, “feel insecure about continuing their university studies year after year”, “consider not continuing their university studies”, and “intend to drop out of university”. The response choices for each item are structured on a 5-point Likert scale ranging from 1 (never) to 5 (always). The reliability and validity of the Italian version of the Intentions to Persist Versus Drop Out scale have been demonstrated in a previous study (Biasi et al., 2017).

### 2.3. Procedure

This study was performed following the recommendations of the Ethical Code of the Italian Association of Psychology (AIP). All subjects agreed to participate in the research project and were granted their written informed consent following the Declaration of Helsinki (2013). The protocol was approved by the Ethics Committee of the Centre for Research and Psychological Intervention (CERIP) of the University of Messina (protocol number: 30465). Only participants who returned signed informed consent forms were allowed to participate in this study. Subsequently, the participants completed the questionnaires in a single session. The questionnaires were administered from October 2022 to September 2023. The privacy and anonymity of their answers were guaranteed. A participation period between 20 and 30 min was required.

### 2.4. Data Analysis

RStudio with the psych package (Revelle, 2018) was used to conduct descriptive statistics, Cronbach’s alpha, and correlations. RStudio with the lavaan package (Rosseel, 2012) was used to carry out structural equation modeling (SEM) with latent variables. The SEM approach was used because it allows multiple dependent variables to be tested simultaneously and is demonstrated to be superior to traditional univariate and multivariate approaches (Iacobucci et al., 2007; Kline, 2023). SEM also provides the opportunity to specify latent variables rather than measured variables, because measured variables are assumed to be measured without error (Coffman & MacCallum, 2005). SEM with latent variables treats constructs measured by questionnaire as latent variables, and multiple indicators for all the constructs that are evaluated are required. Each latent construct’s parcel consisted of the aggregated mean of group items from the questionnaire items to which participants responded to a common scale. A parcel can be defined as an aggregate-level indicator comprising the sum or average of two or more responses or items (Little et al., 2002). Parcels (group) of items for all the constructs of this research were used as indicators. In accordance with previous studies, we created each parcel by combining the items with the highest and lowest item–total correlations, reflecting a strategy of equalizing the influence of the factor across item parcels. Therefore, in the parceling procedure, item indicators serve as tools that allow one to build a measurement model for a clear latent construct (Bagozzi & Edwards, 1998; Coffman & MacCallum, 2005; Little et al., 2002). We chose to use the parceling procedure because it improves commonality across indicators, reduces random error, increases modeling efficiency, and shows normalized distributions compared to employing individual items and total scale scores (Bagozzi & Edwards, 1998; Coffman & MacCallum, 2005; Hau & Marsh, 2004; Kishton & Widaman, 1994; MacCallum et al., 1999; Matsunaga, 2008). Regarding the model fit, the indexes of model fit are usually more acceptable when parcels, rather than items, are modeled because of the psychometric and estimation advantages of parcels. Reflecting on a strategy of equalizing the influence of the factor across item parcels (Hall et al., 1999), we used confidence intervals of the direct and indirect effects with 5000 bootstrap replication samples. A 95% bias-corrected CI was applied following the recommendations of Wu and Jia (2013), Preacher and Hayes (2008), and Shrout and Bolger (2002). Several indexes of fit were examined: the Chi-square (χ2) value; χ2/df; the comparative fit index (CFI); and the root mean square error of approximation (RMSEA) with its 90% confidence interval (CI) (for a description of these indices, see Hair et al. (2006)). The cut-off for a good model fit is achieved when the CFI and TLI values are >0.90, the GFI and AGFI values are >0.90, and the RMSEA is <0.08. Gender and age were also included in this model as control variables.

## 3. Results

The internal consistency of the questionnaires was assessed using Cronbach’s alpha (α). Cronbach’s alpha for the single variables of the PALS indicated good reliability (*Mastery Goal Orientation:* α = 0.86; *Performance-Avoidance Orientation:* α = 0.83; and *Performance-Approach Orientation:* α = 0.90). The MBI-SS subscales showed good and acceptable reliability (*Emotional Exhaustion:* α = 0.85; *Cynicism:* α = 0.85; and *Professional Efficacy:* α = 0.75). Lastly, the Intentions to Persist Versus Drop Out scale exhibited excellent reliability with a Cronbach’s alpha of 0.93.

The correlations presented in Table 1 reveal significant relationships between all the dimensions studied. Mastery goals are positively correlated with professional efficacy (0.595) and negatively correlated with emotional exhaustion (−0.324), cynicism (−0.557), and academic dropout intentions (−0.472). In contrast, PAp goals show a negative correlation with professional efficacy (−0.085) and a positive correlation with emotional exhaustion (0.276), cynicism (0.343), and academic dropout intentions (0.217). PAv goals are negatively correlated with professional efficacy (−0.221), while they are positively correlated with emotional exhaustion (0.394), cynicism (0.438), and academic dropout intentions (0.371). Emotional exhaustion is positively correlated with academic dropout intentions (0.515). Similarly, cynicism shows a positive correlation with academic dropout intentions (0.660).

Structural Equation Modeling (SEM) with latent variables was employed to investigate the mediating role of academic burnout (professional efficacy, cynicism, and emotional exhaustion) in the association between achievement goals (mastery goals, PAv goals, and PAp goals) and ID. An estimation of this model yielded a good fit [χ2 (131) = 844.319, *p* = 0.000, CFI = 0.96, SRMR = 0.04, RMSEA (90%CI) = 0.06 (0.056, 0.064), TLI = 0.95, GFI = 0.94, AGFI = 0.92].

**Figure 1:**
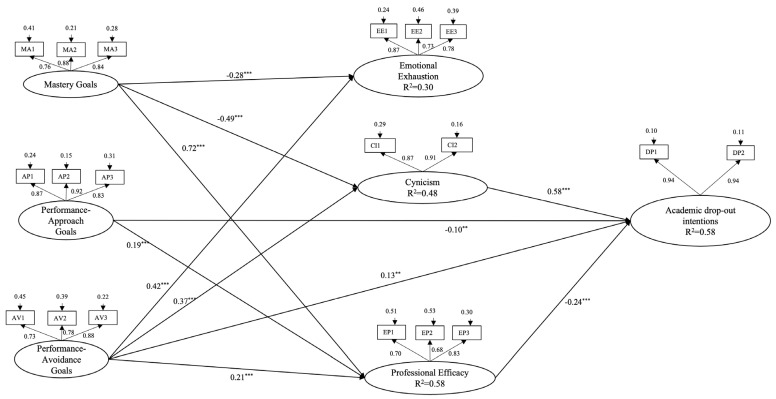
A full mediation model. The coefficients shown are standardized direct path coefficients. The insignificant paths have not been inserted. The coefficients’ correlations are as follows: mastery goals <--> PAp goals: −0.17 ***; mastery goals <--> PAv goals: −0.27 ***; PAp goals <--> PAv goals: 0.71 ***; emotional exhaustion <--> cynicism: 0.66 ***; emotional exhaustion <--> professional efficacy: −0.27 ***. Note: *** *p* ≤ 0.001, ** *p* ≤ 0.01.

The results (Figure 1) show that emotional exhaustion was negatively predicted by mastery goals (β = −0.28, *p* ≤ 0.001) and positively by PAv goals (β = 0.42, *p* ≤ 0.001). Cynicism was negatively predicted by mastery goals (β = −0.49, *p* ≤ 0.001) and positively by PAv goals (β = 0.37, *p* ≤ 0.001). Professional efficacy was positively predicted by mastery goals (β = 0.72, *p* ≤ 0.001) and PAp goals (β = 0.19, *p* ≤ 0.001), and negatively by PAv goals (β =- 0.21, *p* ≤ 0.001). ID was positively predicted by cynicism (β = 0.58, *p* ≤ 0.001) and PAv goals (β = 0.13, *p* ≤ 0.01) and negatively predicted by professional efficacy (β = −0.24, *p* ≤ 0.001) and PAp goals (β = −0.10, *p* ≤ 0.01). Examining the indirect effects, from achievement goals to ID, highlights some significant indirect effect (Table 2): from mastery goals to ID via cynicism (β = 0.28, *p* ≤ 0.001); from PAv goals to ID via cynicism (β = 0.21, *p* ≤ 0.001); from mastery goals to ID via professional efficacy (β = −0.18, *p* ≤ 0.001); from PAp goals to ID via professional efficacy (β = −0.04, *p* ≤ 0.001); and from PAp goals to ID via professional efficacy (β = 0.05, *p* ≤ 0.001). The SEM analysis showed that the indicators were significant for each latent variable, with scores ranging from 0.73 to 0.92.

## 4. Discussion

Despite previous studies (Abreu Alves et al., 2022; Altharman et al., 2023; Tuominen-Soini et al., 2012; Usán Supervía & Salavera Bordás, 2020) highlighting the relation between students’ achievement goals, academic burnout, and the intention to drop out, to date, no study has identified the role of academic burnout as a mediator between student achievement goals and ID in a sample of university students. Therefore, this study aimed to provide preliminary support for the indirect relationship between student achievement goals and academic dropout intentions through the mediating role of academic burnout (emotional exhaustion, cynicism, and professional efficacy). We expected achievement goals to be related to all dimensions of academic burnout, thus increasing or reducing academic dropout intentions.

As displayed in the correlation matrix, all dimensions of goal orientation (mastery goals, PAv goals, and PAp goals) are correlated with dimensions of burnout (emotional exhaustion, cynicism, and professional efficacy) and academic dropout intentions. This finding further underscores the significant relationship between the type of goals students set for themselves and their propensity to experience emotional exhaustion and cynicism or professional efficacy. These factors are also associated with intentions to persist or discontinue academic pursuits prior to the completion of studies. The results obtained from the SEM with latent variables partially support our hypotheses.

From the analysis of direct relationships, it can be observed that mastery and PAv goals are linked to all three dimensions of burnout (emotional exhaustion, cynicism, and professional efficacy), while PAp goals are linked only to the professional efficacy dimension. This highlights the importance of considering the relationship between the different reasons of *why* and *how* students engage in a task and the emotional, physical, and mental reactions to overwhelming educational demands. Consistent with the previous literature (Elliot & Church, 1997; Erfani & Maleki, 2015; Fazli & Fouladchang, 2019; Nadon et al., 2020; Poorgholamy et al., 2020; Usán Supervía & Salavera Bordás, 2020), which highlights how the desire to acquire knowledge and skills may be a protective factor against the risk of burnout, our results underline that students who pursue mastery goals may feel less emotionally exhausted, less cynical, and more efficient professionally. These results can be explained by the fact that students pursuing mastery goals believe that their intelligence can be increased, do not worry excessively about possible failure, and believe that they possess the basic skills to succeed effectively academically and cope with the demands of the university environment, consequently showing fewer symptoms of burnout (Pouratashi & Zamani, 2020).

In contrast, students pursuing performance goals, rather than being driven by the desire to increase their skills for themselves, focus on the desire to demonstrate their skills to others. The way students try to demonstrate their skills over others (PAv or PAp goals) reveals different relationships with burnout dimensions. Our results indicate that students who declare to try to avoid looking incompetent compared to others (PAv goals) might experience more emotional exhaustion and cynicism and less professional efficacy. Conversely, students who affirm to try to surpass others and prove their competence (PAp goals) might feel more professionally efficient and may not show symptoms of emotional exhaustion and cynicism. These results are comparable to those reported by Naidoo et al. (2012) in a study on a sample of U.S. college students, which confirms the relationship between PAv goals and emotional exhaustion. Students who are afraid of appearing incompetent compared to others, trying to avoid negative judgments, may feel drained by academic demands and thus perceive themselves as physically and emotionally drained of their energy (Isoard-Gautheur et al., 2016; Naidoo et al., 2012; Pouratashi & Zamani, 2020). Although Naidoo et al. (2012) did not demonstrate a relationship between PAv goals and cynicism, our results, in agreement with those of Zahed et al. (2014), identify how students who pursue PAv goals might have a more cynical attitude toward their studies. Those who pursue PAv goals might experience feelings of detachment and negativity toward the academic environment and study tasks, which they perceive as a threat to their sense of competence. In addition, several studies have indicated that avoidance goals, characterized by the deliberate evasion of unfavorable outcomes or circumstances, may foster the establishment of a detrimental cycle. This cycle begins with low professional efficacy or a perceived lack of competence in their role, which frequently prompts individuals to adopt avoidance goals as a coping mechanism. These goals, however, tend to prioritize the minimization of failure or the avoidance of challenging tasks rather than engaging with them proactively. This pattern of avoidance, in turn, could perpetuate a downward spiral, leading to a further diminution of professional efficacy and the exacerbation of feelings of incompetence or inadequacy. This, in turn, heightens the risk of experiencing academic burnout. The negative feedback loop created by this cycle can make it more challenging for individuals to break free from it because their avoidance strategies may prevent them from addressing the root causes of stress and dissatisfaction in their studies. This could lead to a continual decline in both motivation and well-being (Ahn & Kim, 2006; Sadoughi & Eskandari, 2024; Son & Lee, 2012). Furthermore, our results agree with those of Naidoo et al. (2012) regarding the relationship between PAp goals and burnout. Naidoo and colleagues noted that students driven by the desire to surpass others and to prove themselves as more competent than others (PAp goals) may experience a greater sense of professional efficacy and thus achieve better academic results. Our study also shows that there are no significant relationships between PAp goals and the other two dimensions of burnout (emotional exhaustion and cynicism). These results can be explained by considering that students who pursue PAp goals might be characterized by a propensity to persist in tasks and a high desire to prove themselves as competent. This may lead them to believe that they can adequately respond to overwhelming academic demands (Honicke et al., 2019).

Moreover, few studies have investigated how students’ goal orientations can play a key role in the premature termination of their studies (Remedios & Richardson, 2013; Senko et al., 2011). Our results highlight that PAv goals are positively related to ID, while PAp goals are negatively related to ID. Conversely, no significant relationship emerged between mastery goals and ID. These findings are in line with those of Remedios and Richardson (2013) and Senko et al. (2011), who emphasized that because students pursuing PAv goals are less intrinsically motivated, they experience less interest and enjoyment in courses of study and thus tend to be more at risk of dropping out of university. Conversely, students who pursue PAp goals, because they want to demonstrate their competencies, may be more likely to continue in the course of study to continue demonstrating their abilities to others, thus proving themselves competent in achieving the degree (Remedios & Richardson, 2013; Senko et al., 2011). The absence of relationships between mastery goals and dropout intentions in our study confirms the results of Remedios and Richardson (2013), who pointed out that it is only the avoidance subcomponent of mastery goals that plays an important role in the ID. However, no significant relationships were found between the approach subcomponent of mastery goals and ID. Our study, considering the trichotomous model of Elliot and Church (1997) as the most widely used model for young adult students (Utman, 1997), considers the mastery dimension as undivided.

The risk of dropping out of academics and exhibiting a lack of consistency in their academic pursuits may be facilitated by burnout (Schaufeli et al., 2002). Our research shows that cynicism is positively related to ID, whereas professional efficacy is negatively related to ID. However, no significant relationship emerged between emotional exhaustion and ID. Although several studies have confirmed an association between burnout and dropout intentions (Abreu Alves et al., 2022; Altharman et al., 2023; Dyrbye et al., 2010; Marôco et al., 2020; Mostert & Pienaar, 2020), to the best of our knowledge, the only study that has examined the association between single dimensions of burnout and ID refers to a sample of high school students (Bask & Salmela-Aro, 2013), while studies in a sample of university students are lacking. By considering the three dimensions of burnout separately, our study highlights how cynicism, a subcomponent of burnout, could increase the risk of ID. According to Bask and Salmela-Aro (2013), students with a negative, callous, and detached attitude toward others, who feel increasingly indifferent to their course of study, are more likely to drop out prematurely. Moreover, in the previously mentioned study, no significant relationship emerged between either the professional efficacy component or the emotional exhaustion component and dropout intentions, as in our study. The findings of our study indicate that students who demonstrate high productivity and effectiveness in their academic activities, along with engagement in study-related behaviors (i.e., professional efficacy), might be less likely to encounter conditions that may foster the ID. The lack of a relationship between emotional exhaustion and ID, which emerged in our study, could be explained in agreement with Bask and Salmela-Aro (2013) and Remedios and Richardson (2013), who presented emotional exhaustion as an individually developed, internalized state of mind, whereas a cynical attitude is described as a state of mind actively directed toward school. Therefore, while individuals with a cynical attitude may actively respond to stressful situations by leaving school, students who experience emotional exhaustion may react passively to stressful situations and do not initiate active withdrawal from school (Bask & Salmela-Aro, 2013; Remedios & Richardson, 2013).

Our study highlights the mediating role of the cynicism and professional efficacy dimensions of academic burnout in the relationship between achievement goals and academic dropout intentions. In particular, based on the perceptions of the students, we observed that mastery goals are negatively linked to the risk of ID through both cynicism and professional efficacy. This result can be explained by considering that students with mastery goals believe that intelligence can be increased. They experience intrinsic pleasure related to learning, which leads them to develop less detachment toward the course of study (cynicism—interpersonal dimension of burnout), reducing the risk of early dropout (Remedios & Richardson, 2013). At the same time, because these students are motivated to develop their skills and perceive challenges as opportunities for growth, they do not worry about possible failures. For this reason, they may experience a high sense of efficacy, competence, and productivity (professional efficacy—self-evaluative dimension of burnout). Concurrently, students who are motivated by a desire to prove themselves as more competent than others (PAp goals) may experience a sense of professional accomplishment and control over their work, confirming themselves as being more effective in their academic performance than others (professional efficacy), which may reduce the likelihood of ID.

In contrast, our study highlights how PAv goals are linked to academic dropout intentions through both cynicism and professional efficacy. As confirmed by the literature, students characterized by a desire to avoid a task for fear of failure and low competence expectancy compared to others (PAv goals) might develop a detachment toward the course of study and self-assess themselves as being incompetent, unproductive, and low-performing. This might promote the ID from the course of study (Bask & Salmela-Aro, 2013; Remedios & Richardson, 2013).

## 5. Limitations and Future Directions

Several limitations should be addressed in future studies. Firstly, this is a cross-sectional study, which does not allow for causal associations. To comprehensively address this issue, future research could adopt a longitudinal approach, utilizing the application of daily diaries (Lim et al., 2024a, 2024b) to allow students to monitor themselves over time, thereby elucidating the causal role of burnout dimensions (such as emotional exhaustion) on PAv goals and the subsequent academic dropout risk. Furthermore, the convenience sample recruited from university students and the lack of an even distribution of male and female participants may limit the generalizability of our findings. Moreover, because the majority of the sample we recruited is from Southern Italy (66.3%), we cannot generalize the results of this study (Seong et al., 2023). Despite the disparities undoubtedly present between Northern, Central, and Southern Italy, the challenges related to the educational system and the academic demands that Italian students face during their university journey are universal, as is also the phenomenon of academic dropout. Nevertheless, future research could expand the sample nationwide to confirm the validity of these results in different contexts. Another limitation is the use of student self-reported questionnaires. Future studies could consider additional methods of data collection, such as direct observation, in addition to self-reports. Regarding cultural factors, such as family pressure and social expectations, future research may benefit from integrating cultural and socioeconomic variables. Although the existing literature indicates that cultural factors may exert an influence on student motivation (Cho et al., 2023; Lee et al., 2023; Lee et al., 2023), this study nevertheless offers an important perspective on intrinsic motivation and burnout. This may prove useful for the design of psychological interventions, such as motivational training. Future research that delves more profoundly into these aspects could provide a more comprehensive and contextualized understanding of the dropout phenomenon. Moreover, it must be mentioned that the professional efficacy dimension of academic burnout had an acceptable reliability (α = 0.75). While the Cronbach’s alphas are moderate, this result may be consistent with the complexity of the construct being measured. The moderate values reflect the multidimensional nature of academic burnout, capturing its distinct yet interconnected dimensions.

Despite these limitations, our study extends the present literature, which is scarce to date, on the relationship between achievement goals, academic burnout, and academic dropout intentions in a sample of university students. To the best of our knowledge, this is one of the first studies to explore the mediating role of academic burnout between these variables among university students. Our study revealed that mastery goal- and PAp goal-oriented students are more likely to self-evaluate effectively and be less detached from studying, which may reduce the risk of dropout. It would be advantageous to implement interventions that foster motivation in students by emphasizing their sense of mastery and demonstrating greater competence. This approach could help reduce students’ feelings of detachment from their academic pursuits, thereby lowering the risk of early academic dropout (Kaplan & Maehr, 2007). This may impact students’ well-being and academic success, increasing their academic satisfaction, sense of efficacy, personal accomplishment, academic engagement, and other emotional, cognitive, and behavioral components that may limit their possible dropout intentions.

In addition, future research should expand on our study by considering different individual and contextual variables that may influence the phenomenon investigated. In particular, it would be appropriate to investigate the same relationships in different academic fields of study, considering dispositional variables (e.g., grit, optimism, and perfectionism) and contextual factors (e.g., academic atmosphere, workload, attending lessons, respecting deadlines, balancing university and private life, teacher support, peer relationships, and family dynamics) (Choi et al., 2024; Seong et al., 2024).

## 6. Conclusions

In conclusion, the results of our study reinforce the important role of achievement goals in dropout intentions. Furthermore, our research emphasizes that the cynicism and professional efficacy dimensions of burnout play a key mediating role in understanding the relationship between achievement goals and academic dropout intentions. The results of our study may be attributed to the fact that the interpersonal and self-evaluative dimensions of burnout are most prevalent among university students (Almalki et al., 2017; Boni et al., 2018; Pala, 2012). Finally, the current study highlights how different motivations for students to pursue different study goals (mastery, PAv, and PAp goals) might play different roles in various dimensions of burnout and early dropout. However, it would be interesting for future studies to further investigate these relationships which, to date, have been poorly examined among university students.

## Figures and Tables

**Table 1:** Correlation matrix.

	1		2		3		4		5		6		7	
1. Mastery goals	—													
2. PAp goals	−0.205	***	—											
3. PAv goals	−0.261	***	0.643	***	—									
4. Emotional exhaustion	−0.324	***	0.276	***	0.394	***	—							
5. Cynicism	−0.557	***	0.343	***	0.438	***	0.635	***	—					
6. Professional efficacy	0.595	***	−0.085	***	−0.221	***	−0.355	***	−0.424	***	—			
7. Academic dropout intentions	−0.472	***	0.217	***	0.371	***	0.515	***	0.66	***	−0.473	***	—	

Note: *** *p* < 0.001.

**Table 2:** Path estimates, SEs, and 95% CIs.

	*β*	*SE*	*Lower Bound (BC)* *95% CI*	*Upper Bound (BC)* *95% CI*	*p*
*Direct Effect*					
Mastery Goals → Emotional exhaustion	−0.28	0.07	−0.79	−0.49	≤0.001
PAv Goals → Emotional exhaustion	0.42	0.08	0.57	0.92	≤0.001
Mastery Goals → Cynicism	−0.49	0.07	−10.0	−0.49	≤0.001
PAv Goals → Cynicism	0.37	0.08	0.52	0.84	≤0.001
Mastery Goals → Professional efficacy	0.72	0.04	10.0	0.72	≤0.001
PAp Goals → Professional efficacy	0.19	0.03	0.08	0.20	≤0.001
PAv Goals → Professional efficacy	−0.21	0.04	−0.30	−0.21	≤0.001
Cynicism → Academic dropout intentions	0.58	0.04	0.37	0.55	≤0.001
Professional Efficacy → Academic dropout intentions	−0.24	0.06	−0.48	−0.22	≤0.001
PAp Goals → Academic dropout intentions	−0.10	0.03	−0.18	−0.03	≤0.01
PAv Goals → Academic dropout intentions	0.13	0.06	0.06	0.31	≤0.01
*Indirect effect* via *Cynicism*					
Mastery Goals → Academic dropout intentions	−0.28	0.06	−0.66	−0.41	≤0.001
PAv Goals → Academic dropout intentions	0.21	0.04	0.22	0.41	≤0.001
*Indirect effect* via *Professional Efficacy*					
Mastery Goals → Academic dropout intentions	−0.18	0.06	−0.45	−0.20	≤0.001
PAp Goals → Academic dropout intentions	−0.04	0.01	−0.07	−0.02	≤0.001
PAv Goals → Academic dropout intentions	0.05	0.02	0.04	0.12	≤0.001

## Data Availability

The original contributions presented in this study are included in the article; further inquiries can be directed to the corresponding authors.

## References

[B1-ejihpe-15-00003] Aarkrog V., Wahlgren B. (2022). Goal orientation and decision-making in education. Vocations and Learning.

[B2-ejihpe-15-00003] Aarkrog V., Wahlgren B., Larsen C. H., Mariager-Anderson K., Gottlieb S. (2018). Decision-making processes among potential dropouts in vocational education and training and adult learning. International Journal for Research in Vocational Education and Training.

[B3-ejihpe-15-00003] Abdulghani H. M., Alanazi K., Alotaibi R., Alsubeeh N. A., Ahmad T., Haque S. (2023). Prevalence of potential dropout thoughts and their influential factors among saudi medical students. Sage Open.

[B4-ejihpe-15-00003] Abreu Alves S., Sinval J., Lucas Neto L., Marôco J., Gonçalves Ferreira A., Oliveira P. (2022). Burnout and dropout intention in medical students: The protective role of academic engagement. BMC Medical Education.

[B5-ejihpe-15-00003] Ahn D., Kim O. (2006). Perfectionism, achievement goals, and academic efficacy in medical students. Korean Journal of Medical Education.

[B6-ejihpe-15-00003] Alivernini F., Manganelli S., Lucidi F. (2018). Personal and classroom achievement goals: Their structures and relationships. Journal of Psychoeducational Assessment.

[B7-ejihpe-15-00003] Almalki S. A., Almojali A. I., Alothman A. S., Masuadi E. M., Alaqeel M. K. (2017). Burnout and its association with extracurricular activities among medical students in Saudi Arabia. International Journal of Medical Education.

[B8-ejihpe-15-00003] Almeida A. N. d., Neres I. V., Nunes A., Souza C. V. N. d. (2019). Effectiveness of public university expansion in Brazil: Comparison between the situation of graduated and dropout students. Ensaio: Avaliação e Políticas Públicas Em Educação.

[B9-ejihpe-15-00003] Altharman H. A., Alnaqi R. I., Buanz S. F., Alsenayien A. Y., Siraj R. A. (2023). Exploring the relationship between burnout, resilience, and dropout intention among nursing students during clinical training in Saudi Arabia. SAGE Open Nursing.

[B10-ejihpe-15-00003] Ambiel R. A. (2015). Construção da escala de motivos para evasão do ensino superior. Avaliação Psicológica.

[B11-ejihpe-15-00003] Ames C. (1992). Classrooms: Goals, structures, and student motivation. Journal of Educational Psychology.

[B12-ejihpe-15-00003] Atkinson J. W. (1964). An introduction to motivation..

[B13-ejihpe-15-00003] Bagozzi R. P., Edwards J. R. (1998). A general approach for representing constructs in organizational research. Organizational Research Methods.

[B14-ejihpe-15-00003] Bardach L., Oczlon S., Pietschnig J., Lüftenegger M. (2020). Has achievement goal theory been right? A meta-analysis of the relation between goal structures and personal achievement goals. Journal of Educational Psychology.

[B15-ejihpe-15-00003] Bask M., Salmela-Aro K. (2013). Burned out to drop out: Exploring the relationship between school burnout and school dropout. European Journal of Psychology of Education.

[B16-ejihpe-15-00003] Bereby-Meyer Y., Kaplan A. (2005). Motivational influences on transfer of problem-solving strategies. Contemporary Educational Psychology.

[B17-ejihpe-15-00003] Bianchi R., Truchot D., Laurent E., Brisson R., Schonfeld I. S. (2014). Is burnout solely job-related? A critical comment. Scandinavian Journal of Psychology.

[B18-ejihpe-15-00003] Biasi V., Vincenzo C. D., Patrizi N. (2017). Relazioni tra autoregolazione dell’apprendimento, motivazioni e successo accademico degli studenti. Identificazione di fattori predittivi del rischio di drop-out. Italian Journal of Educational Research.

[B19-ejihpe-15-00003] Boekaerts M., Urdan T. C., Karabenick S. A. (2010). Motivation and self-regulation: Two close friends. Advances in motivation and achievement.

[B20-ejihpe-15-00003] Boni R. A. d. S., Paiva C. E., De Oliveira M. A., Lucchetti G., Fregnani J. H. T. G., Paiva B. S. R. (2018). Burnout among medical students during the first years of undergraduate school: Prevalence and associated factors. PLoS ONE.

[B21-ejihpe-15-00003] Bresó E., Schaufeli W. B., Salanova M. (2011). Can a self-efficacy-based intervention decrease burnout, increase engagement, and enhance performance? A quasi-experimental study. Higher Education.

[B22-ejihpe-15-00003] Bzuneck J. A., Boruchovitch E. (2019). Motivação de estudantes no ensino superior: Como fortalecê-la?. Estudantes Do Ensino Superior: Desafios e Oportunidades.

[B23-ejihpe-15-00003] Caballero C. C., Esteve E. B., Gutierréz O. G. (2015). Burnout in university students. Psicología Desde El Caribe.

[B24-ejihpe-15-00003] Cellar D. F., Stuhlmacher A. F., Young S. K., Fisher D. M., Adair C. K., Haynes S., Twichell E., Arnold K. A., Royer K., Denning B. L., Riester D. (2011). Trait Goal orientation, self-regulation, and performance: A meta-analysis. Journal of Business and Psychology.

[B25-ejihpe-15-00003] Chambel M. J., Curral L. (2005). Stress in academic life: Work characteristics as predictors of student well-being and performance. Applied Psychology.

[B26-ejihpe-15-00003] Cho S., Lee M., Lee S. M. (2023). Burned-out classroom climate, intrinsic motivation, and academic engagement: Exploring unresolved issues in the job demand-resource model. Psychological Reports.

[B27-ejihpe-15-00003] Choi H., Cho S., Kim J., Lee S. M. (2024). The role of teacher support in the self-esteem of Korean adolescents with burnout. Journal of Psychologists and Counsellors in Schools.

[B28-ejihpe-15-00003] Church M. A., Elliot A. J., Gable S. L. (2001). Perceptions of classroom environment, achievement goals, and achievement outcomes. Journal of Educational Psychology.

[B29-ejihpe-15-00003] Cobo-Rendón R., Hojman V., García-Álvarez D., Cobo Rendon R. (2023). Academic emotions, college adjustment, and dropout intention in university students. Frontiers in Education.

[B30-ejihpe-15-00003] Coffman D. L., MacCallum R. C. (2005). Using parcels to convert path analysis models into latent variable models. Multivariate Behavioral Research.

[B31-ejihpe-15-00003] Dalbosco S. N. P., Ferraz A. S., dos Santos A. A. A. (2018). Metas de realização, autorregulação da aprendizagem e autopercepção de desempenho em universitários. Revista Brasileira de Orientação Profissional.

[B32-ejihpe-15-00003] Darnon C., Butera F., Mugny G., Quiamzade A., Hulleman C. S. (2009). “Too complex for me!” Why do performance-approach and performance-avoidance goals predict exam performance?. European Journal of Psychology of Education.

[B33-ejihpe-15-00003] Dweck C. S. (1986). Motivational processes affecting learning. American Psychologist.

[B34-ejihpe-15-00003] Dweck C. S., Leggett E. L. (1988). A social-cognitive approach to motivation and personality. Psychological Review.

[B35-ejihpe-15-00003] Dykman B. M. (1998). Integrating cognitive and motivational factors in depression: Initial tests of a goal-orientation approach. Journal of Personality and Social Psychology.

[B36-ejihpe-15-00003] Dyrbye L. N., Thomas M. R., Power D. V., Durning S., Moutier C., Massie F. S. J., Harper W., Eacker A., Szydlo D. W., Sloan J. A., Shanafelt T. D. (2010). Burnout and serious thoughts of dropping out of medical school: A multi-institutional study. Academic Medicine.

[B37-ejihpe-15-00003] (2024). Early leavers from education and training.

[B38-ejihpe-15-00003] Elliot A. J. (1999). Approach and avoidance motivation and achievement goals. Educational Psychologist.

[B39-ejihpe-15-00003] Elliot A. J. (2005). A conceptual history of the achievement goal construct. Handbook of competence and motivation.

[B40-ejihpe-15-00003] Elliot A. J., Church M. A. (1997). A hierarchical model of approach and avoidance achievement motivation. Journal of Personality and Social Psychology.

[B41-ejihpe-15-00003] Elliot A. J., Harackiewicz J. M. (1996). Approach and avoidance achievement goals and intrinsic motivation: A mediational analysis. Journal of Personality and Social Psychology.

[B43-ejihpe-15-00003] Elliott E. S., Dweck C. S. (1983). Achievement motivation. Handbook of child psychology: Social and personality development.

[B42-ejihpe-15-00003] Elliott E. S., Dweck C. S. (1988). Goals: An approach to motivation and achievement. Journal of Personality and Social Psychology.

[B44-ejihpe-15-00003] Erfani N., Maleki H. (2015). Predicting academic burnout based on attribution styles and goal orientation of female students. International Journal of Innovation and Research in Educational Sciences.

[B45-ejihpe-15-00003] Fazli A., Fouladchang M. (2019). The relation of academic conscience to academic burnout: The mediating role of academic goal orientation. Journal of Education Strategies in Medical Sciences.

[B46-ejihpe-15-00003] Fiorilli C., De Stasio S., Di Chiacchio C., Pepe A., Salmela-Aro K. (2017). School burnout, depressive symptoms and engagement: Their combined effect on student achievement. International Journal of Educational Research.

[B47-ejihpe-15-00003] Graham S., Golan S. (1991). Motivational influences on cognition: Task involvement, ego involvement, and depth of information processing. Journal of Educational Psychology.

[B48-ejihpe-15-00003] Hair J. F., Black W. C., Babin B. J., Anderson R. E., Tatham R. L. (2006). Multivariate data analysis.

[B49-ejihpe-15-00003] Hall R. J., Snell A. F., Foust M. S. (1999). Item parceling strategies in SEM: Investigating the subtle effects of unmodeled secondary constructs. Organizational Research Methods.

[B50-ejihpe-15-00003] Hardre P. L., Reeve J. (2003). A motivational model of rural students’ intentions to persist in, versus drop out of, high school. Journal of Educational Psychology.

[B51-ejihpe-15-00003] Hau K., Marsh H. W. (2004). The use of item parcels in structural equation modelling: Non-normal data and small sample sizes. British Journal of Mathematical and Statistical Psychology.

[B52-ejihpe-15-00003] Honicke T., Broadbent J., Fuller-Tyszkiewicz M. (2019). Learner self-efficacy, goal orientation, and academic achievement: Exploring mediating and moderating relationships. Higher Education Research & Development.

[B53-ejihpe-15-00003] Howell A. J., Watson D. C. (2007). Procrastination: Associations with achievement goal orientation and learning strategies. Personality and Individual Differences.

[B54-ejihpe-15-00003] Hulleman C. S., Schrager S. M., Bodmann S. M., Harackiewicz J. M. (2010). A meta-analytic review of achievement goal measures: Different labels for the same constructs or different constructs with similar labels?. Psychological Bulletin.

[B55-ejihpe-15-00003] Iacobucci D., Saldanha N., Deng X. (2007). A meditation on mediation: Evidence that structural equations models perform better than regressions. Journal of Consumer Psychology.

[B56-ejihpe-15-00003] IsHak W., Nikravesh R., Lederer S., Perry R., Ogunyemi D., Bernstein C. (2013). Burnout in medical students: A systematic review. The Clinical Teacher.

[B57-ejihpe-15-00003] Isoard-Gautheur S., Trouilloud D., Gustafsson H., Guillet-Descas E. (2016). Associations between the perceived quality of the coach–athlete relationship and athlete burnout: An examination of the mediating role of achievement goals. Psychology of Sport and Exercise.

[B58-ejihpe-15-00003] Kaplan A., Maehr M. L. (2007). The contributions and prospects of goal orientation theory. Educational Psychology Review.

[B59-ejihpe-15-00003] Kaplan A., Middleton M. J., Urdan T., Midgley C. (2002). Achievement goals and goal structures. Goals, goal structures, and patterns of adaptive learning.

[B60-ejihpe-15-00003] Kishton J. M., Widaman K. F. (1994). Unidimensional versus domain representative parceling of questionnaire items: An empirical example. Educational and Psychological Measurement.

[B61-ejihpe-15-00003] Kline R. B. (2023). Principles and practice of structural equation modeling.

[B62-ejihpe-15-00003] Lee M., Lee T., Lee S. M. (2023). Role of peer support in competitive classroom climates: Focusing on the mediation effect of academic hatred in the JD-R model. Journal of Psychologists and Counsellors in Schools.

[B63-ejihpe-15-00003] Lee T., Hong S. E., Kang J., Lee S. M. (2023). Role of achievement value, teachers’ autonomy support, and teachers’ academic pressure in promoting academic engagement among high school seniors. School Psychology International.

[B64-ejihpe-15-00003] Lim H., Jang G., Park G., Lee H., Lee S. M. (2024a). Impact of state and trait emotion regulation on daily emotional exhaustion among Korean school counsellors. Stress and Health.

[B65-ejihpe-15-00003] Lim H., Lee T., Weng C., Lee S. M. (2024b). Effects of surface acting on exhaustion of Korean school counselors. Journal of Counseling & Development.

[B66-ejihpe-15-00003] Linnenbrink E. A., Pintrich P. R. (2000). Multiple pathways to learning and achievement: The role of goal orientation in fostering adaptive motivation, affect, and cognition. Intrinsic and extrinsic motivation.

[B67-ejihpe-15-00003] Little T. D., Cunningham W. A., Shahar G., Widaman K. F. (2002). To parcel or not to parcel: Exploring the question, weighing the merits. Structural Equation Modeling: A Multidisciplinary Journal.

[B68-ejihpe-15-00003] Liu W. C. (2021). Implicit theories of intelligence and achievement goals: A Look at students’ intrinsic motivation and achievement in mathematics. Frontiers in Psychology.

[B69-ejihpe-15-00003] Liu Z., Xie Y., Sun Z., Liu D., Yin H., Shi L. (2023). Factors associated with academic burnout and its prevalence among university students: A cross-sectional study. BMC Medical Education.

[B70-ejihpe-15-00003] Lorenzo-Quiles O., Galdón-López S., Lendínez-Turón A. (2023). Factors contributing to university dropout: A review. Frontiers in Education.

[B71-ejihpe-15-00003] MacCallum R. C., Widaman K. F., Zhang S., Hong S. (1999). Sample size in factor analysis. Psychological Methods.

[B72-ejihpe-15-00003] Madigan D. J., Curran T. (2021). Does burnout affect academic achievement? A meta-analysis of over 100,000 students. Educational Psychology Review.

[B73-ejihpe-15-00003] Marôco J., Assunção H., Harju-Luukkainen H., Lin S.-W., Sit P.-S., Cheung K., Maloa B., Ilic I. S., Smith T. J., Campos J. A. D. B. (2020). Predictors of academic efficacy and dropout intention in university students: Can engagement suppress burnout?. PLoS ONE.

[B74-ejihpe-15-00003] Maslach C. (2001). What have we learned about burnout and health?. Psychology & Health.

[B75-ejihpe-15-00003] Maslach C., Jackson S. E., Leiter M. P. (1997). Maslach burnout inventory.

[B76-ejihpe-15-00003] Mason H. D., Nel J. A. (2012). Compassion Fatigue, burnout and compassion satisfaction: Prevalence among nursing students. Journal of Psychology in Africa.

[B77-ejihpe-15-00003] Matsunaga M. (2008). Item parceling in structural equation modeling: A primer. Communication Methods and Measures.

[B78-ejihpe-15-00003] McClelland D. C. (1961). Achieving society.

[B79-ejihpe-15-00003] Mega C., Ronconi L., De Beni R. (2014). What makes a good student? How emotions, self-regulated learning, and motivation contribute to academic achievement. Journal of Educational Psychology.

[B80-ejihpe-15-00003] Midgley C., Maehr M. L., Hruda L. Z., Anderman E., Anderman L., Freeman K. E., Urdan T. (2000). Manual for the patterns of adaptive learning scales.

[B81-ejihpe-15-00003] Morelli M., Chirumbolo A., Baiocco R., Cattelino E. (2023). Self-regulated learning self-efficacy, motivation, and intention to drop-out: The moderating role of friendships at University. Current Psychology.

[B82-ejihpe-15-00003] Mostert K., Pienaar J. (2020). The moderating effect of social support on the relationship between burnout, intention to drop out, and satisfaction with studies of first-year university students. Journal of Psychology in Africa.

[B83-ejihpe-15-00003] Nadon L., Babenko O., Chazan D., Daniels L. M. (2020). Burning out before they start? An achievement goal theory perspective on medical and education students. Social Psychology of Education.

[B84-ejihpe-15-00003] Naidoo L. J., DeCriscio A., Bily H., Manipella A., Ryan M., Youdim J. (2012). The 2 × 2 Model of Goal Orientation and Burnout: The Role of Approach–Avoidance Dimensions in Predicting Burnout. Journal of Applied Social Psychology.

[B85-ejihpe-15-00003] Nicholls J. G. (1984). Achievement motivation: Conceptions of ability, subjective experience, task choice, and performance. Psychological Review.

[B86-ejihpe-15-00003] Noh H., Shin H., Lee S. M. (2013). Developmental process of academic burnout among Korean middle school students. Learning and Individual Differences.

[B87-ejihpe-15-00003] OECD (2019). Tertiary graduation rate *[Dataset]*.

[B88-ejihpe-15-00003] Pala A. (2012). The burnout level among faculty of education students at Celal Bayar University. Procedia-Social and Behavioral Sciences.

[B89-ejihpe-15-00003] Peetsma T., Veen I. V. D. (2013). Avoidance-oriented students’ development in motivation for maths, self-regulated learning behaviour and achievement: A person-centred study in the lowest level of secondary education. Educational Psychology.

[B90-ejihpe-15-00003] Pintrich P. R. (2003). A motivational science perspective on the role of student motivation in learning and teaching contexts. Journal of Educational Psychology.

[B91-ejihpe-15-00003] Poorgholamy F., Kazemi S., Barzegar M., Sohrabi N. (2020). Predicting academic burnout based on achievement goals and self-regulated learning. Iranian Evolutionary Educational Psychology Journal.

[B92-ejihpe-15-00003] Portoghese I., Leiter M. P., Maslach C., Galletta M., Porru F., D’Aloja E., Finco G., Campagna M. (2018). Measuring burnout among university students: Factorial validity, invariance, and latent profiles of the italian version of the maslach burnout inventory student survey (MBI-SS). Frontiers in Psychology.

[B93-ejihpe-15-00003] Pouratashi M., Zamani A. (2020). Students’ psychological characteristics and its relationship with exhaustion, cynicism, and academic inefficacy. International Journal of Knowledge and Learning.

[B94-ejihpe-15-00003] Preacher K. J., Hayes A. F. (2008). Asymptotic and resampling strategies for assessing and comparing indirect effects in multiple mediator models. Behavior Research Methods.

[B95-ejihpe-15-00003] Remedios R., Richardson J. T. E. (2013). Achievement goals in adult learners: Evidence from distance education. British Journal of Educational Psychology.

[B96-ejihpe-15-00003] Revelle W. (2018). psych: Procedures for psychological, psychometric, and personality research.

[B97-ejihpe-15-00003] Rosseel Y. (2012). lavaan: An R package for structural equation modeling. Journal of Statistical Software.

[B98-ejihpe-15-00003] Sadoughi M., Eskandari N. (2024). The relationship between achievement goal orientations and academic burnout among medical students: The mediating role of academic grit. Journal of Medical Education Development.

[B99-ejihpe-15-00003] Salmela-Aro K., Vuori J., Vuori J., Blonk R., Price R. H. (2015). School engagement and burnout among students: Preparing for work life. Sustainable working lives.

[B100-ejihpe-15-00003] Schaufeli W. B., Greenglass E. R. (2001). Introduction to special issue on burnout and health. Psychology & Health.

[B101-ejihpe-15-00003] Schaufeli W. B., Martínez I. M., Pinto A. M., Salanova M., Bakker A. B. (2002). Burnout and engagement in university students: A cross-national study. Journal of Cross-Cultural Psychology.

[B102-ejihpe-15-00003] Schauffeli W., Salanova M. (2007). Efficacy or inefficacy, that’s the question: Burnout and work engagement, and their relationships with efficacy believes. Anxiety, Stress and Coping.

[B103-ejihpe-15-00003] Senko C., Hulleman C. S., Harackiewicz J. M. (2011). Achievement goal theory at the crossroads: Old controversies, current challenges, and new directions. Educational Psychologist.

[B104-ejihpe-15-00003] Seong H., Lee S., Lee S. M. (2023). Is autonomy-supportive parenting universally beneficial? Combined effect of socially prescribed perfectionism and parental autonomy support on stress in emerging adults in South Korea. Current Psychology.

[B105-ejihpe-15-00003] Seong H., Lim H., Jang G.-E., Park G., Kang J., Lee S. M. (2024). Relationship between interpersonal emotion regulation and social support and their effects on depressive symptoms in korean emerging adults. Cognitive Therapy and Research.

[B106-ejihpe-15-00003] Shin H., Puig A., Lee J., Lee J. H., Lee S. M. (2011). Cultural validation of the maslach burnout inventory for Korean students. Asia Pacific Education Review.

[B107-ejihpe-15-00003] Shrout P. E., Bolger N. (2002). Mediation in experimental and nonexperimental studies: New procedures and recommendations. Psychological Methods.

[B108-ejihpe-15-00003] Sideridis G. D. (2005). Goal orientation, academic achievement, and depression: Evidence in favor of a revised goal theory framework. Journal of Educational Psychology.

[B109-ejihpe-15-00003] Son J., Lee S. (2012). The effect of perfectionism on job burnout and professional efficacy: A mediation effect of goal orientations. Korean Journal of Industrial and Organizational Psychology.

[B110-ejihpe-15-00003] Tayebi A., Gómez J., Delgado C. (2021). Analysis on the lack of motivation and dropout in engineering students in Spain. IEEE Access.

[B111-ejihpe-15-00003] Tuominen-Soini H., Salmela-Aro K., Niemivirta M. (2012). Achievement goal orientations and academic well-being across the transition to upper secondary education. Learning and Individual Differences.

[B112-ejihpe-15-00003] Usán P., Salavera C., Teruel P. (2019). School motivation, goal orientation and academic performance in secondary education students. Psychology Research and Behavior Management.

[B113-ejihpe-15-00003] Usán Supervía P., Salavera Bordás C. (2020). Burnout, goal orientation and academic performance in adolescent students. International Journal of Environmental Research and Public Health.

[B114-ejihpe-15-00003] Utman C. H. (1997). Performance effects of motivational state: A meta-analysis. Personality and Social Psychology Review.

[B115-ejihpe-15-00003] Vallerand R. J., Fortier M. S., Guay F. (1997). Self-determination and persistence in a real-life setting: Toward a motivational model of high school dropout. Journal of Personality and Social Psychology.

[B116-ejihpe-15-00003] Vandewalle D., Nerstad C. G. L., Dysvik A. (2019). Goal orientation: A review of the miles traveled and the miles to go. Annual Review of Organizational Psychology and Organizational Behavior.

[B117-ejihpe-15-00003] Van Yperen N. W., Elliot A. J., Anseel F. (2009). The influence of mastery-avoidance goals on performance improvement. European Journal of Social Psychology.

[B118-ejihpe-15-00003] Veroff J. (1969). Social comparison and the development of achievement motivation. Achievement-related motives in children.

[B119-ejihpe-15-00003] Wissing M. P., Khumalo I. P., Oosthuizen T. M., Nienaber A., Kruger A., Potgieter J. C., Temane Q. M. (2011). Coping self-efficacy as mediator in the dynamics of psychological well-being in various contexts. Journal of Psychology in Africa.

[B120-ejihpe-15-00003] Wu W., Jia F. (2013). A new procedure to test mediation with missing data through nonparametric bootstrapping and multiple imputation. Multivariate Behavioral Research.

[B121-ejihpe-15-00003] Zahed B. A., Pourbahram R., Rahmani J. S. (2014). The relationship of perfectionism, goal achievement orientation and academic performance to academic burnout.

